# Implementing the Melanocytic Pathology Assessment Tool and Hierarchy for Diagnosis: Long-term effect of a simple educational intervention

**DOI:** 10.1016/j.jdin.2023.01.025

**Published:** 2023-03-12

**Authors:** Lisa M. Reisch, Hannah Shucard, Andrea C. Radick, Megan M. Eguchi, David E. Elder, Raymond L. Barnhill, Michael W. Piepkorn, Stevan R. Knezevich, Kathleen F. Kerr, Joann G. Elmore

**Affiliations:** a Department of Biostatistics, University of Washington, Seattle, Washington; b Department of Medicine, University of California, Los Angeles, David Geffen School of Medicine, Los Angeles, California; c Department of Pathology and Laboratory Medicine, Hospital of the University of Pennsylvania, Philadelphia, Pennsylvania; d Department of Translational Research, Institut Curie, Paris Sciences and Lettres Research University, and University of Paris UFR of Medicine, Paris, France; e Division of Dermatology, Department of Medicine, University of Washington School of Medicine, Seattle, Washington; f Dermatopathology Northwest, Bellevue, Washington; g Pathology Associates, Clovis, California

**Keywords:** continuing medical education, dermatopathology, intervention study, melanocytic skin lesion, standardized histology schema

## Abstract

**Background:**

A standardized pathology management tool for melanocytic skin lesions may improve patient care by simplifying interpretation and categorization of the diverse terminology currently extant.

**Objective:**

To assess an online educational intervention that teaches dermatopathologists to use the Melanocytic Pathology Assessment Tool and Hierarchy for Diagnosis (MPATH-Dx), a schema collapsing multiple diagnostic terms into 5 classes ranging from benign to invasive melanoma.

**Methods:**

Practicing dermatopathologists (*N =* 149) from 40 US states participated in a 2-year educational intervention study (71% response rate). The intervention involved a brief tutorial followed by practice on 28 melanocytic lesions, with the goal of teaching pathologists how to correctly use the MPATH-Dx schema; competence using the MPATH-Dx tool 12-24 months postintervention was assessed. Participants’ self-reported confidence using the MPATH-Dx tool was assessed preintervention and postintervention.

**Results:**

At preintervention, confidence using the MPATH-Dx tool was already high, despite 68% lacking prior familiarity with it, and confidence increased postintervention (*P =* .0003). During the intervention, participants used the MPATH-Dx tool correctly for 90% of their interpretations; postintervention, participants used the MPATH-Dx tool correctly for 88% of their interpretations.

**Limitations:**

Future research should examine implementing a standardized pathology assessment schema in actual clinical practice.

**Conclusion:**

Dermatopathologists can be taught to confidently and competently use the MPATH-Dx schema with a simple educational tutorial followed by practice.

**Capsule Summary**

• The value of a tool to provide standardized classification of the currently diverse pathology terminology for melanocytic skin lesions, to facilitate management, is recognized by dermatopathologists.
• With a simple tutorial, dermatopathologists can confidently and correctly apply the Melanocytic Pathology Assessment Tool and Hierarchy for Diagnosis schema to improve communication and patient care.

## Introduction

A standardized pathology assessment tool for melanocytic skin lesions is not currently mandated for clinical practice but could improve patient care by simplifying interpretation and classification of the diverse terminology currently in use. Although international standards already exist for melanoma, the use of this tool could facilitate greater consistency and higher-quality research into management approaches for benign melanocytic lesions. In 2014, the Melanocytic Pathology Assessment Tool and Hierarchy for Diagnosis (MPATH-Dx) reporting schema was developed to categorize diagnoses of melanocytic skin lesions by mapping the many diverse diagnostic terms into 5 classes, each with corresponding risk assessments and treatment recommendations.^1^ The intention of the MPATH-Dx is not to mandate the replacement of the currently used terminology, but to supplement current systems and to provide a categorization of terms into classes that have similar prognostic and management implications, to simplify treatment decisions, especially for less specialized providers.

The scheme includes melanoma but is expected to be more useful for benign lesions, for which standardized recommendations do not exist, with some limited exceptions.^2^, ^3^, ^4^, ^5^ The MPATH-Dx schema was modeled after the Breast Imaging-Reporting and Data System (BI-RADS), mandated for use in the field of breast imaging.^6^ Over the past decade, the MPATH-Dx has been successfully used in research.^7^, ^8^, ^9^, ^10^, ^11^ The International Melanoma Pathology Study Group has evaluated^12^ and helped refine the MPATH-Dx^13^, the schema is included in dermatopathology textbooks^14^^,^^15^ and mentioned in the World Health Organization Classification of Skin Tumours,^16^^,^^17^ and interest of pathologists around the globe has been piqued.^18^ US dermatopathologists have recognized the value of a standardized system for categorizing melanocytic skin lesions for patient management and research, and have reported willingness to embrace the MPATH-Dx concept in their own practices to improve communication and patient care.^19^

The teachability of the MPATH-Dx schema has not yet been studied in a sample of practicing dermatopathologists. We developed a simple online MPATH-Dx educational tutorial to train pathologists, provided participants with an opportunity to practice what they had learned, and evaluated the effect of this educational intervention on the confidence and correct use of the MPATH-Dx tool. We hypothesized that pathologists would gain confidence and sustained competence using this tool due to this educational intervention.

## Materials and methods

The educational intervention took place within the larger Reducing Errors in Melanocytic Interpretations study, which has been described in detail elsewhere.^10^^,^^20^ In brief, potential study participants were identified in 40 geographically diverse states, using a list of board-certified dermatopathologists from Direct Medical Data, LLC databases. Potential participants were contacted by email (maximum of 3 attempts), which was followed by telephone calls (maximum of 2 attempts) and postal mail (1 attempt) to verify eligibility. Eligible participants met the following criteria: board-certified and/or fellowship training in dermatopathology, currently practicing in the United States, interpreted melanocytic skin biopsies within the previous year, and were expected to continue interpreting melanocytic skin lesions for the next 2 years. Dermatopathologists verified as eligible (*N* = 226) were invited to enroll in the study between July 2018 and July 2019. Data collection continued through May 2021.

All procedures were Health Insurance Portability and Accountability Act (HIPAA) compliant, and approval was obtained from the Institutional Review Boards of the Fred Hutchinson Cancer Research Center in Seattle, WA, and the David Geffen School of Medicine at the University of California Los Angeles. Participants who completed all aspects of the study received up to 25 AMA Category 1 Continuing Medical Education Credits.

### Study cases

Cutaneous melanocytic lesions from the shave, punch, and excisional specimens were gathered from a pacific northwest laboratory and then prepared onto hematoxylin and eosin stained glass slides. Cases were selected using stratification based on patient age and medical chart documentation of the original diagnosis.^11^ Three experienced dermatopathologists independently reviewed each case, followed by consensus review using a modified Delphi approach, to arrive at a consensus reference diagnosis for each case.^21^ The study cases were divided into 5 slide sets of 28 cases each, with intentionally higher proportions of MPATH-Dx classes II-V than are typically encountered in clinical practice. All participants were randomly assigned to interpret a slide set of 28 cases in both phases I and II.

### MPATH-Dx schema

The MPATH-Dx schema has been described in full detail elsewhere.^1^ In brief, the MPATH-Dx schema comprises a histology reporting form and a mapping tool. The MPATH-Dx schema allows users to translate many diagnostic terms to 5 MPATH-Dx classes, a process that we refer to as “mapping.” The MPATH-Dx classes range from benign (class I) to invasive melanoma (class V), and each class is associated with suggested treatments. The following are examples of diagnostic terms within each class and suggested treatment recommendations: Class I (e.g., nevus/mild atypia, no further treatment required); class II (e.g. moderate atypia, narrow but complete re-excision <5mm); class III (e.g., severe atypia/melanoma *in situ*, repeat excision with ≥5mm but <1cm margins), class IV (e.g., T1a invasive melanoma, wide excision ≥1cm) and class V (e.g., ≥T1b invasive melanoma, wide excision ≥1cm with possible additional treatment; e.g., sentinel lymph node biopsy and adjuvant interferon therapy).

### Study procedures

**Fig 1:**
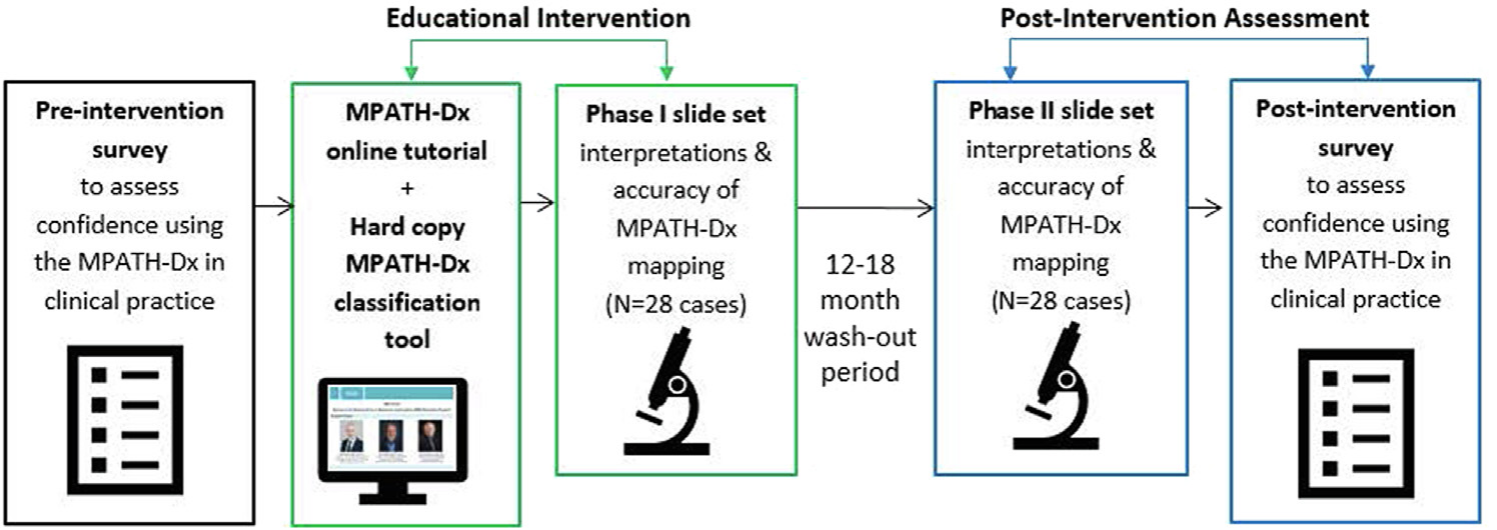
Flowchart of Preintervention survey, Educational intervention, and Postintervention assessment procedures.

The MPATH-Dx Educational Intervention Study (Fig 1) included 3 components: (1) Preintervention survey; (2) Educational intervention, and (3) Postintervention assessment.

#### Preintervention survey

Participating dermatopathologists completed a brief online preintervention survey, which gathered demographic information and queried participants on their familiarity with the MPATH-Dx classification tool. Participants were asked “A 2014 publication in the Journal of the American Academy of Dermatology by Piepkorn et al^1^ describes in detail the MPATH-Dx classification tool participants will use in this educational program. Are you already familiar with the MPATH-Dx classification tool?” and were given the opportunity to check “no”, “yes”, or “unsure.” Because participants had not necessarily engaged with the MPATH-Dx before the preintervention survey, participants were then shown a summary of the MPATH-Dx schema and were queried about their confidence using a standardized taxonomy for the field. Participants rated “How confident would you be in using the MPATH-Dx classification tool in clinical practice?” on a 6-point scale ranging from “1: Not at all confident” to “6: Very confident.” Following completion of the survey, participants selected a one-week window to begin the educational component.

#### Educational intervention

##### MPATH-Dx tutorial

During the one-week window of their choosing, participants were mailed a hard copy of the MPATH-Dx schema to aid in their learning and for reference during the tutorial and slide reviews (Supplementary Material, available via Mendeley at https://data.mendeley.com/datasets/cxkv26n853/1). Participants reviewed a 30-minute online tutorial on all MPATH-Dx classes (class I -benign through class V -invasive melanoma), including instructions on how to map their diagnostic interpretations into these classes, followed by case examples and exercises to ensure understanding.

##### Phase 1 slide review

A slide set of 28 melanocytic lesion glass slides was included in the mailing along with the aforementioned hard copy of the MPATH-Dx schema; case selection and slide sets have been described in detail elsewhere.^8^^,^^20^ After completing the MPATH-Dx tutorial, participants reviewed each case and entered their diagnostic interpretations for each case into an online version of the MPATH-Dx histology reporting form. For each of the 28 cases, participants then answered the question “Which MPATH-Dx class would you classify this case into?” by choosing the appropriate MPATH-Dx class for the case. The slide review gave participants the opportunity to practice using what they had learned from the MPATH-Dx tutorial on a variety of different classes of cases. This was not a diagnostic accuracy study, but instead examined participants’ competence with self-mapping their diagnoses into the correct MPATH-Dx classes.

#### Postintervention assessment

##### Phase II slide review

After a wash-out period of 12-24 months (mean of 16 months) at a convenient one-week window of their choosing, participants were mailed a slide set of 28 cases along with another hard copy of the MPATH-Dx tool. Of the 28 cases, 18 were identical to cases interpreted in phase 1, and 10 were new cases. Using identical methods to phase I interpretations, participants documented their interpretations for each case and mapped their diagnoses using the MPATH-Dx schema, as described above. The slide review served to assess retention of the correct use of the MPATH-Dx schema.

##### Postintervention survey

Following completion of their phase 2 slide review, participants completed an online postintervention survey. Participants were asked “Now that you have completed the Reducing Errors in Melanocytic Interpretations Study and CME, how confident are you in using the MPATH-Dx classification tool?” on a 6-point scale ranging from “Not at all confident” (1) to “Very confident” (6).

## Results

**Fig 2:**
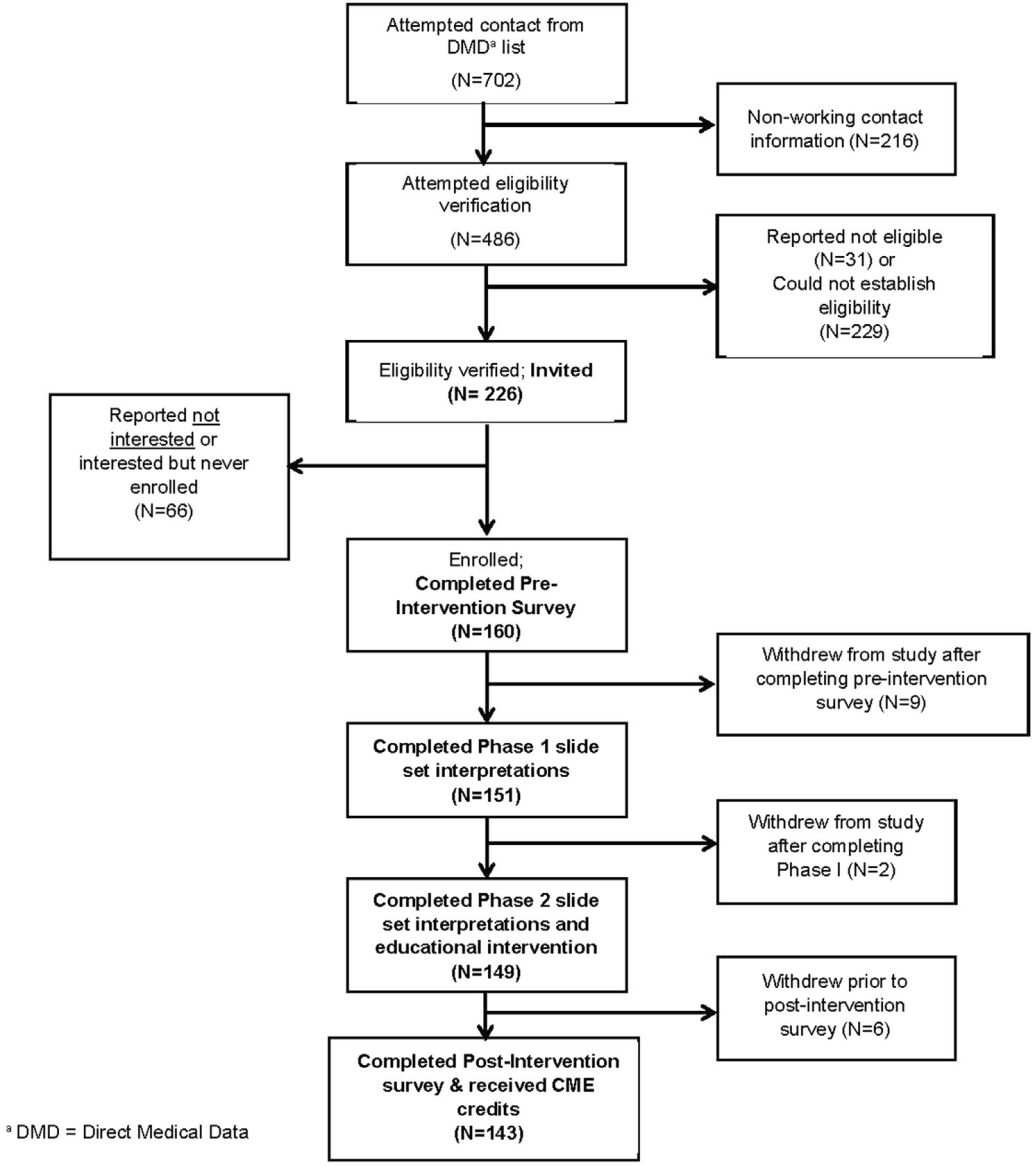
Participant recruitment and participant flow chart. *DMD*, Direct medical data.

Of 226 invited dermatopathologists, 160 consented and completed the preintervention survey (response rate = 71%), 151 of those completed the educational intervention, and 143 of those completed all components of the study, including the postintervention assessment (study retention rate = 89%, see Fig 2). In phase I, a total of 4,228 diagnostic interpretations and mapped MPATH-Dx classifications were gathered from the 151 participants who completed the educational intervention. In phase II, an additional 4,172 diagnostic interpretations and mapped MPATH-Dx classifications were gathered from the 149 participants who completed the phase II slide sets.

Demographic and experience characteristics of participating dermatopathologists are depicted in Table I. Participants were broadly distributed geographically around the U.S., as well as among age groups, years interpreting melanocytic skin lesions, and percentage of caseload interpreting melanocytic skin lesions. Slightly over half reported an affiliation with an academic medical center with either a primary (21%) or adjunct/affiliate appointment (31%). As expected given eligibility criteria, 100% were board-certified and/or fellowship training in dermatopathology, with 99% of participants having both credentials.

**Table I:** Demographic and experience characteristics of dermatopathologists (N=149)

Pathologist characteristics	*N* (%)
Demographics	
Age (y)	
<40	26 (17%)
40–49	64 (43%)
50–59	39 (26%)
≥60	20 (13%)
Sex	
Female	46 (31%)
Male	101 (68%)
Prefer not to answer	2 (1%)
Geographical region∗	
Northeast	29 (19%)
Midwest	44 (30%)
Southern	61 (41%)
Western	15 (10%)
Training and experience	
Affiliation with an academic medical center	
No	71 (48%)
Yes, adjunct/affiliated	46 (31%)
Yes, primary appointment	32 (21%)
Residency†	
Anatomic pathology	26 (17%)
Anatomic/Clinical pathology	81 (54%)
Dermatology	50 (34%)
Other‡	3 (2%)
Fellowship†	
No fellowship	2 (1%)
Surgical pathology	29 (19%)
Dermatopathology	147 (99%)
Other§	11 (7%)
Board certification†	
Not board-certified	0 (0%)
Dermatology	49 (33%)
Anatomic pathology	107 (72%)
Clinical pathology	77 (52%)
Dermatopathologyװ	148 (99%)
Other¶	10 (7%)
Years of interpreting melanocytic skin lesions	
<5	18 (12%)
5–9	38 (26%)
10–19	62 (42%)
≥20	31 (21%)
Percent of caseload interpreting melanocytic skin lesions	
<10%	7 (5%)
10%-24%	72 (48%)
25%-49%	52 (35%)
≥50%	18 (12%)
Are you already familiar with the MPATH-Dx classification tool?	
No	80 (54%)
Unsure	21 (14%)
Yes	48 (32%)

*MPATH-Dx*, Melanocytic Pathology Assessment Tool and Hierarchy for Diagnosis.

∗ US Census Bureau Regions: https://www.census.gov/prod/1/gen/95statab/preface.pdf.

† Pathologists could make multiple selections; therefore, percentages may sum to greater than 100%.

‡ ‘Other’ includes residencies in internal medicine and general surgery.

§ ‘Other’ includes the following responses for fellowship training: cytopathology, gastrointestinal and Hepatic pathology, hematology, hematopathology, molecular pathology, soft tissue pathology, and transfusion medicine.

װ One participant reported fellowship training in dermatopathology but was not board-certified in dermatopathology.

¶ ‘Other’ includes the following responses for board certification: cytopathology, DI/DLI, forensic pathology, hematology, hematopathology, internal medicine, and transfusion medicine.

**Fig 3:**
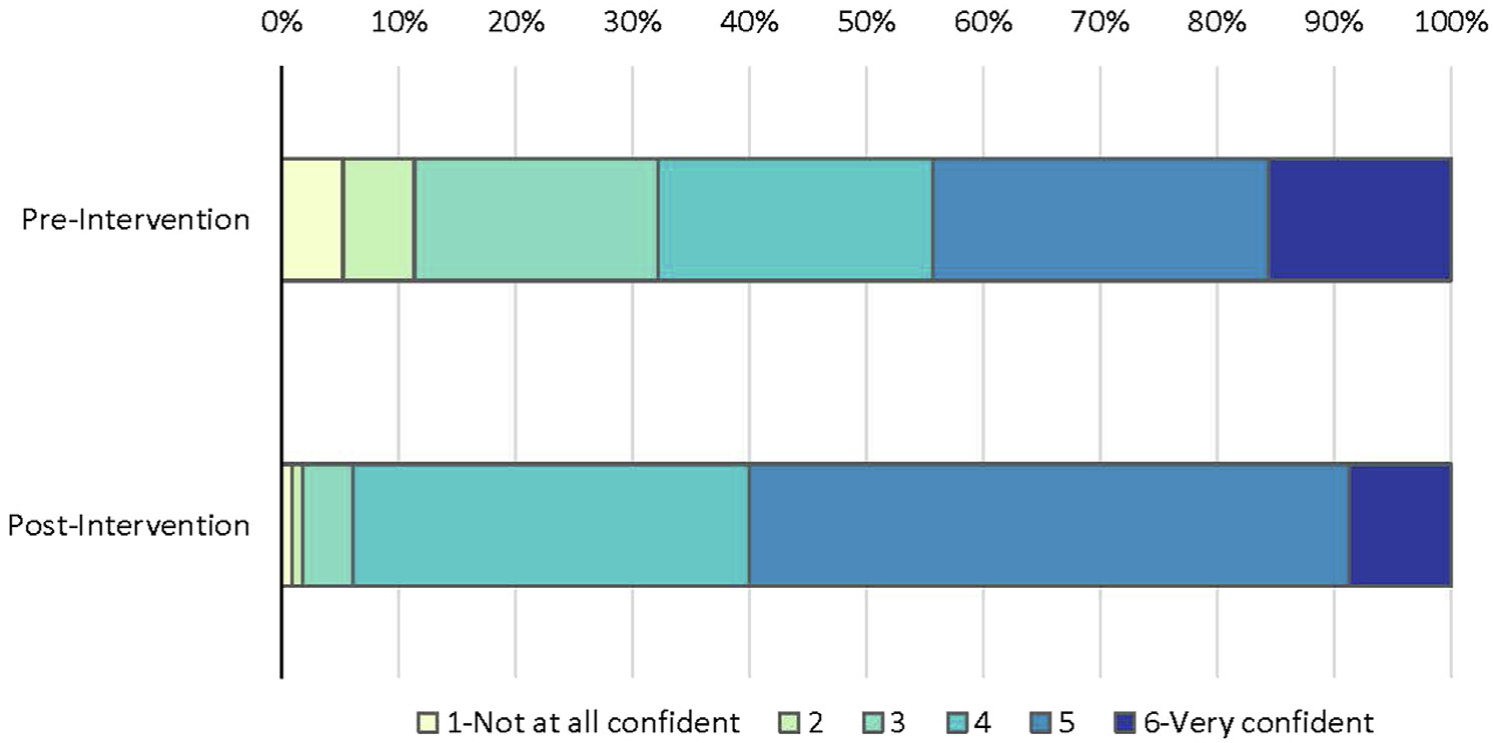
Participant confidence in using the MPATH-Dx classification tool in clinical practice. Using the 6-point Likert scale, mean confidence was 4.1 on the preintervention survey and 4.6 on the postintervention survey (N = 115 participants). *MPATH-Dx*, Melanocytic Pathology Assessment Tool and Hierarchy for Diagnosis.

**Fig 4:**
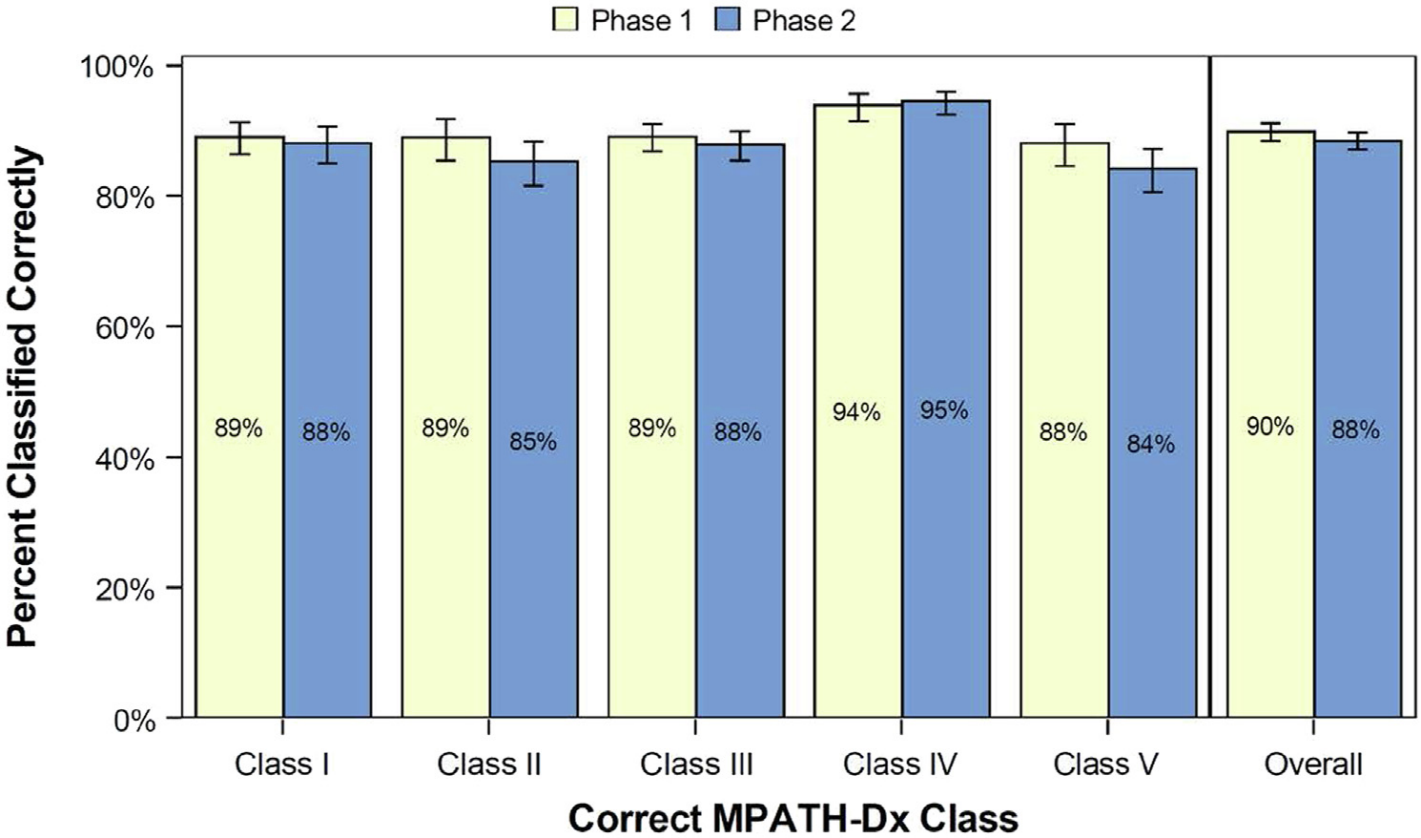
Demonstrated competence of all study participants (N = 149) using the MPATH-Dx soon after the educational tutorial (phase I) and after approximately 12 to 18 months (phase II). Bars represent 95% CIs. In total, 149 participants interpreted 4,172 cases in phase I and 4,172 cases in phase II. *MPATH-Dx*, Melanocytic Pathology Assessment Tool and Hierarchy for Diagnosis.

At the preintervention survey, 68% of participants reported a lack of familiarity with the MPATH-Dx schema (or were unsure), and yet after reading a summary of the schema, their self-reported perceived confidence in being able to use the MPATH-Dx classification tool was already high **(**mean= 4.1 on 6-point Likert scale, Fig 3). Perceived confidence increased by postintervention assessment (mean= 4.6; *P =* .0003). At the phase I slide set interpretations, immediately after viewing the tutorial, participants correctly used the MPATH-Dx tool for 90% of their interpretations overall. At phase II slide set interpretations, 12-24 months after initially completing the educational tutorial, participants still correctly used the MPATH-Dx tool for 88% of their interpretations. Competence using the MPATH-Dx classification tool is shown for both phase I and phase II assessments in Figure 4.

## Discussion

In a study of diagnostic terminology used in current practice, it was found that pathologists use a wide range and diversity of terminology when interpreting melanocytic neoplasms^12^, potentially compromising the quality of care and providing support for the use of a simplified pathology assessment tool. Dermatopathologists who participated in this 2-year study demonstrated high competence in correctly using the MPATH-Dx tool. Importantly, this competence was sustained 12-24 months after exposure to the educational tutorial. Participants also reported high confidence using the tool, which increased over time.

Our prior work documented that dermatopathologists recognize the value of using a standardized lexicon of terminologies for melanocytic skin lesions and express a willingness to embrace such a scheme in their clinical practice.^19^ The current study builds on this by showing that dermatopathologists are easily able to follow the instructions of a pathology assessment tool to correctly map their diagnostic interpretations into a standardized format. Evidenced by the success of similar classification tools in other specialities (e.g., the Breast Imaging-Reporting and Data System system^6^) as well as support from the International Melanoma Pathology Study Group (IMPSG)^12^, and expressed interest by other research teams^18^, the field appears poised to embrace and implement a standardized schema, such as the MPATH-Dx, for assistance in developing management strategies, for classifying melanocytic cases in studies, and for improving patients’ and clinicians’ understanding of pathologists’ diagnoses and suggested treatments. Our findings support a simple educational method for familiarizing dermatopathologists with a management-related classification schema, for which competence is quickly gained in applying it.

This study had several limitations. With survey research, there is always the possibility of socially -desirable responses. However, confidence ratings are difficult or impossible to ascertain by a mechanism other than self-report. We did not gather data on participants’ time spent practicing using the MPATH-Dx tool in clinical practice between phases I and II, nor did we require such practice. Continued practice during the wash-out period between phases I and II may have increased their mastery of the tool to an even greater degree, but we did not gather the data to address that possibility. We also did not gather data on what classification systems, if any, participants were using in practice before this study. This study was performed outside clinical practice and limited to US dermatopathologists only; future research on the implementation of a standardized taxonomy within the context of actual clinical practice will be necessary, as well as including international practicing dermatopathologists. It is possible that some participants misunderstood the histology form question “Which MPATH-Dx class would you classify this case into,” and mistakenly interpreted the question as MPATH-Dx class selection being their own prerogative despite the fact that we referred them to the hard copy of the MPATH-Dx (diagnostic-treatment mapping) classification tool. Finally, the MPATH-Dx schema was recently revised into an MPATH-Dx 2.0 version, whereby most moderate atypias do not require a re-excision.^13^ As new data emerges^22^ and the field evolves, the MPATH-Dx (and the educational tutorial) will need to be updated accordingly.

The most important study strength is the long-term follow-up of participants after they were involved in the educational intervention. Most educational intervention studies in pathology have been described as having poor experimental design, including fewer than 100 study participants, lacking data collection among multiple institutions, and failing to provide statistical significance.^23^ No medical education intervention studies in a recent review article examined outcomes at a distance from the intervention.^23^ The need for an evidence base in the practice of medical education is essential^24^ and our study establishes an evidence base for the MPATH-Dx schema, including a large nationwide sample of pathologists from multiple institutions and establishing long-term outcomes of the intervention with statistical significance and confidence intervals provided. Other study strengths include a high response rate (71%) among invited dermatopathologists, which surpasses standards for physician surveys.^25^, ^26^, ^27^ We also reported a high retention rate from enrollment to completion (89%), which is quite remarkable given the required time commitment of many hours over 2 years. Participants were all experienced board-certified and/or fellowship-trained dermatopathologists, representing both academic and non-academic affiliations and geographically diverse locations throughout the US, supporting the generalizability of our findings. The educational tutorial was developed by the research team who developed and studied the MPATH-Dx classification tool over the past decade, ensuring that the nuances of the schema were adequately addressed and properly instructed.

## Conclusion

We propose that the implementation of a management-related pathology assessment schema in routine practice may provide a robust tool for standardized diagnostic reporting of melanocytic lesions and management of patients. This functions as a supplement to the extant user-generated diagnostic nomenclatures and taxonomy, with the goal of benefiting both health care providers and patients. Such a schema is only useful if dermatopathologists can be taught to use it correctly. The findings herein demonstrate that when the field is poised to implement a standardized management-related system, dermatopathologists can be taught to use the MPATH-Dx reporting schema correctly with a simple educational tutorial followed by practice.

## Conflicts of interest

None disclosed.
